# Spontaneous Neural Activity in the Superior Temporal Gyrus Recapitulates Tuning for Speech Features

**DOI:** 10.3389/fnhum.2018.00360

**Published:** 2018-09-18

**Authors:** Jonathan D. Breshears, Liberty S. Hamilton, Edward F. Chang

**Affiliations:** ^1^Department of Neurosurgery, University of California, San Francisco, San Francisco, CA, United States; ^2^Department of Communication Sciences and Disorders, University of Texas at Austin, Austin, TX, United States; ^3^Department of Neurology, Dell Medical School, University of Texas at Austin, Austin, TX, United States

**Keywords:** electrocorticography (ECoG), speech perception, high gamma activity, resting state networks, spatiotemporal dynamics

## Abstract

**Background:** Numerous studies have demonstrated that individuals exhibit structured neural activity in many brain regions during rest that is also observed during different tasks, however it is still not clear whether and how resting state activity patterns may relate to underlying tuning for specific stimuli. In the posterior superior temporal gyrus (STG), distinct neural activity patterns are observed during the perception of specific linguistic speech features. We hypothesized that spontaneous resting-state neural dynamics of the STG would be structured to reflect its role in speech perception, exhibiting an organization along speech features as seen during speech perception.

**Methods:** Human cortical local field potentials were recorded from the superior temporal gyrus (STG) in 8 patients undergoing surgical treatment of epilepsy. Signals were recorded during speech perception and rest. Patterns of neural activity (high gamma power: 70–150 Hz) during rest, extracted with spatiotemporal principal component analysis, were compared to spatiotemporal neural responses to speech features during perception. Hierarchical clustering was applied to look for patterns in rest that corresponded to speech feature tuning.

**Results:** Significant correlations were found between neural responses to speech features (sentence onsets, consonants, and vowels) and the spontaneous neural activity in the STG. Across subjects, these correlations clustered into five groups, demonstrating tuning for speech features—most robustly for acoustic onsets. These correlations were not seen in other brain areas, or during motor and spectrally-rotated speech control tasks.

**Conclusions:** In this study, we present evidence that the RS structure of STG activity robustly recapitulates its stimulus-evoked response to acoustic onsets. Further, secondary patterns in RS activity appear to correlate with stimulus-evoked responses to speech features. The role of these spontaneous spatiotemporal activity patterns remains to be elucidated.

## Introduction

Direct cortical recordings from frontal, parietal, and occipital regions in humans and primates have shown that spontaneous neural oscillations are spatiotemporally structured into networks, present during task performance, rest, sleep, and anesthesia (Vincent et al., 2007; He et al., 2008; Breshears et al., 2010; Foster et al., 2015; Chelaru et al., 2016; Gratton et al., 2018). While this work has demonstrated convincingly that individuals exhibit structured activity during rest that is also observed during different tasks, it is still not clear whether and how resting state activity patterns may relate to underlying tuning for specific stimuli.

In the posterior superior temporal gyrus (pSTG), distinct neural activity patterns are observed during the perception of specific linguistic speech features (Chang et al., 2010; Leaver and Rauschecker, 2010; Steinschneider et al., 2011; Friederici, 2012; Mesgarani et al., 2014; Berezutskaya et al., 2017; Hamilton et al., 2018) and information about the pitch of a speaker's voice separately from phonetic content (Tang et al., 2017). More specifically, the parcellation of human pSTG into a posterior onset and an anterior sustained zone for speech (Hamilton et al., 2018) posits the existence of separate networks for distinct temporal information in speech, but it is not clear whether this parcellation would also be observed at rest, when no stimuli are present. Recent animal work has shown that spontaneous RS neural activity in macaque auditory cortex recapitulates the tonotopic organization seen during auditory stimulus presentation, suggesting that specific neural tuning is evident during rest (Fukushima et al., 2012). Similarly, in macaque visual cortex patterns of spontaneous neural activity have been shown to recapitulate stimulus-evoked retinotopy (Lewis et al., 2016).

A better understanding of how tuning for speech stimuli is recapitulated in the structure of spontaneous neural activity is essential for our understanding of speech perception and language development. Before a stimulus is presented, an initial resting-state is the context from which the neural process of speech perception begins. Before neural tuning to speech stimuli has developed, speech perception and production must be learned in the context of resting-state. This understanding is also important for clinical applications such as the development of speech and auditory neuroprostheses. We hypothesized that spontaneous resting-state neural dynamics of STG would be structured to reflect its role in speech perception, exhibiting an organization along speech features as seen during speech perception.

## Methods

### Subjects

This study was carried out in accordance with the recommendations of the Human Research Protection Program of the University of California San Francisco Office of Ethics and Compliance. The study protocol was approved by the University of California, San Francisco Institutional Review Board. All subjects gave written informed consent in accordance with the Declaration of Helsinki. The study included neurosurgical patients undergoing craniotomy for placement of subdural electrodes for seizure focus localization as part of treatment for intractable epilepsy. Patients with electrode coverage of the STG were eligible for the study.

Eight right-handed, left language dominant subjects were included in the study, 4 with right hemisphere coverage and 4 with left hemisphere coverage. There were 4 women, and the median age was 30.5 years. Subject demographics, clinical information, and electrode coverage is shown in Table 1.

**Table 1:** Subject demographics, clinical information, and electrode coverage.

**Subject**	**Age/sex**	**Hemisphere with electrodes**	**Functional language testing**	**Seizure focus/Resection site**	**Cognitive deficits**	**Number of electrodes**				
						**STG**	**MTG**	**SMC**	**IFG**	**SM**
R1	32F	Right	MEG	Superior frontal gyrus	Attention	40	35	41	30	20
R2	30M	Right	None	Anterior temporal lobe	Attention, processing speed, and executive function deficits	39	0	119	8	76
R3	20M	Right	MEG	Anterior temporal lobe	None	45	29	59	30	8
R4	47F	Right	Wada	Mesial temporal lobe	None	47	39	72	33	23
L5	31F	Left	MEG	Mesial temporal lobe	Slow cognitive process and verbal memory	60	14	76	23	42
L6	45M	Left	Wada	Posterior STG	Subtle verbal memory impairment	60	32	75	24	27
L7	29F	Left	MEG	Anterolateral and subtemporal cortex	None	60	32	80	34	15
L8	28M	Left	MEG	Hippocampus	None	70	19	89	6	47

*STG, superior temporal gyrus; MTG, middle temporal gyrus; SMC, sensorimotor cortex (pre- and post-central gyrus); IFG, inferior frontal gyrus; SMG, supramarginal gyrus; MEG, magnetoencephalogram*.

### Data collection and preprocessing

#### Neural recordings

Local field potential signals were recorded from 256-channel electrode grids (AdTech or Integra) with 1.17 mm exposed contact size and 4 mm spacing, at a sampling rate of 3051.8 Hz using a 256-channel PZ2 amplifier or a 512-channel PZ5 amplifier connected to an RZ2 digital acquisition system (Tucker-Davis Technologies, Alachua, FL, USA). We recorded the local field potential from each electrode and applied a notch filter at 60 Hz and its harmonics at 120 and 180 Hz to reduce line-noise related artifacts. In addition, signals from each electrode were visually inspected for artifact, which was then removed from further analysis. A common average reference was then applied across all electrodes. A Hilbert transform was used to extract the power in the high gamma frequency band (70–150 Hz) using procedures described previously (Moses et al., 2016). High gamma signals were then down-sampled to 100 Hz. Signals from each electrode were z-scored individually within each experimental block, by subtracting the mean, then dividing by the standard deviation.

#### Electrode localization

We localized the electrodes on each participant's brain by co-registering the pre-operative T1 MRI scan to the postoperative CT scan containing the electrode locations. Pial surfaces were reconstructed using FreeSurfer routines. Anatomic labels were assigned to each electrode by finding the closest surface vertex (according to Euclidean distance) and determining the FreeSurfer label of that point from the annotation file associated with the Desikan-Killiany atlas. Finally, a quality check was done on the automated anatomic labeling to confirm no electrodes were mislabeled. Full electrode localization procedures are described in Hamilton et al. (2017). The electrode coverage and anatomic labeling for each subject is shown in Figure S1.

#### Stimuli

Electrical activity was recorded from the 256-contact subdural electrode arrays under two conditions. In the first “speech perception” condition, subjects passively listened to 499 unique sentences from the TIMIT corpus, presented in 5 min blocks of 100–124 sentences each. Most sentences were repeated 2 times, and a subset of 10 sentences was repeated 10 or 20 times each. Sentences were presented in pseudorandom order with 0.4 s of silence in between each sentence. Participants completed between 5 and 12 5-min blocks, where blocks were recorded across several days of each participant's hospital stay. If blocks occurred during the same recording session, participants would generally take a break of 1–2 min in between consecutive blocks. The stimuli were presented through two stereo free-field speakers (Logitech) at a distance of 4 feet from the subject (Garofolo et al., 1993). The sentences were spoken by 286 male and 116 female talkers from different regions of the United States. Task presentation was controlled using custom MATLAB® (MathWorks®, Natick, MA) code on a Windows laptop.

In the second “resting state” condition, patients were asked to sit silent and motionless, with their eyes open or closed, while data was collected. The resting state recordings were similarly performed over several days of the participants' hospital stay, often but not always occurring on the same days as the speech perception condition. Resting state activity was generally recorded in 30 s to 1 min blocks. If the subject was noted to have fallen asleep this was noted by the experimenter and the data was not used. The stimulus presentation and/or ambient noise was recorded with a microphone (Sennheiser e845S) in both conditions for quality control. Microphone recordings were reviewed to ensure there was silence during the “resting state” condition and no contaminating noise during “passive listening” to TIMIT sentences. Segments of noise contamination (i.e., talking or acoustic noise in the room) were removed from analysis. All subsequent analysis was performed in MATLAB® using custom routines.

#### Spatiotemporal correlation of spontaneous and speech evoked HGP in STG

**Figure 1:**
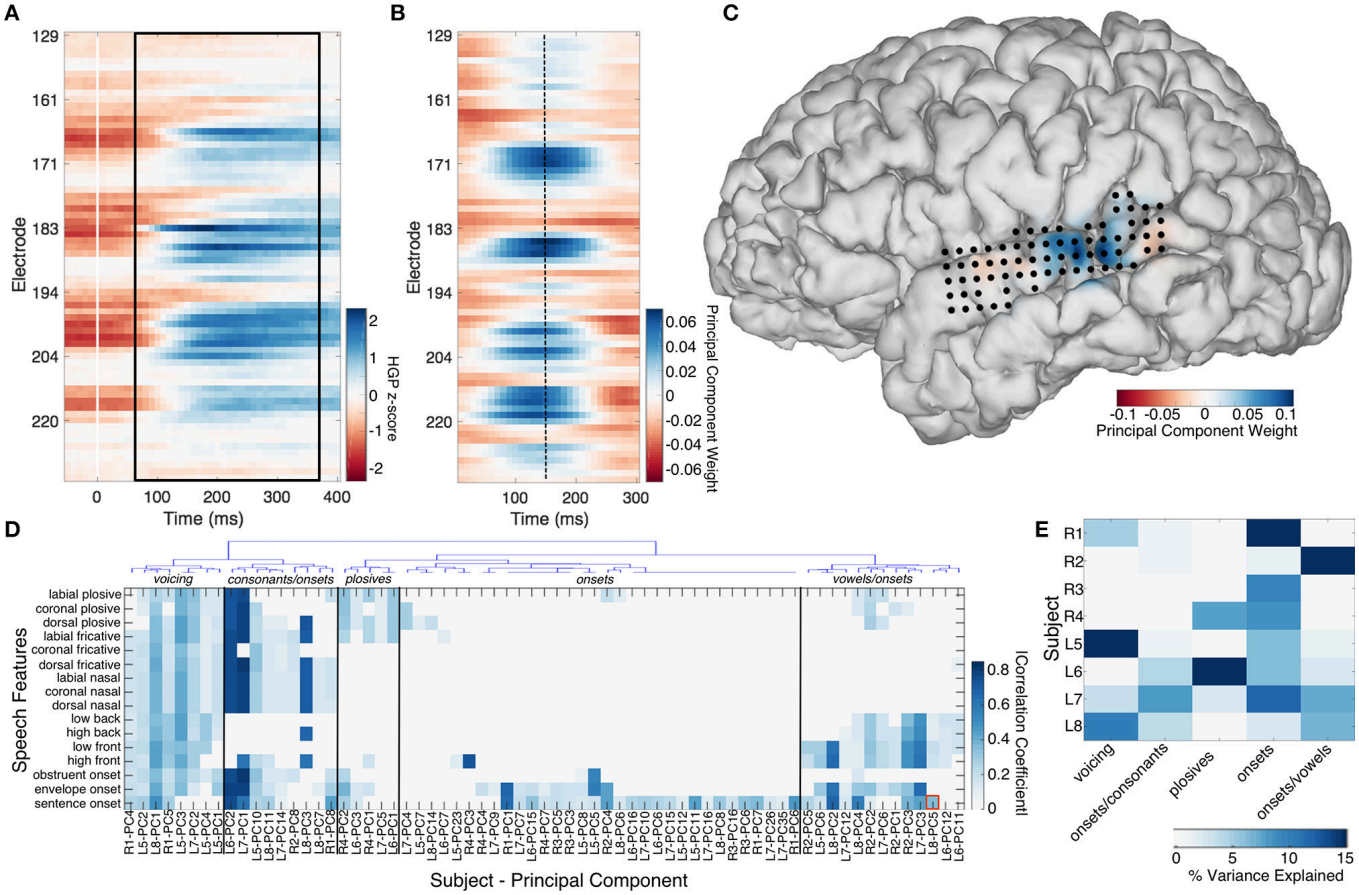
The median high gamma spatiotemporal response to sentence onsets is shown across 70 STG electrodes in subject L8 **(A)**. Trials were aligned to the stimulus onset (time = 0). The black box denotes the 300 ms epoch with significant correlation (*R* = 0.36) to a spatiotemporal principal component trained on resting state activity from the same 70 electrodes, shown in **(B)**. Electrode locations on STG are shown in **(C)**, superimposed over the principal component weights plotted for a single time point (dashed line at 150 ms in **B**). Hierarchical clustering of the composite correlation results from 8 subjects are shown in **(D)**. Resting state stPCs correlating with speech feature evoked response patterns clustered into 5 groups, denoted by the blue linkage tree (top) and vertical black lines. These clusters roughly correspond to five distinct speech feature groups (black labels across the top: “voicing,” “consonants/onsets,” “plosives,” “onsets,” and “vowels/onsets”). The red box denotes the correlation coefficient for the example shown in **(A–C)**. The percentage of the resting state data variance explained by stPCs significantly correlating with speech evoked patterns is shown in **(E)**.

The median high gamma spatiotemporal response of STG, middle temporal gyrus (MTG), sensorimotor cortex (SMC), inferior frontal gyrus (IFG), and supramarginal gyrus (SMG) to 16 speech features was calculated by using the TIMIT linguistic transcription to align the high gamma power (HGP) signals from the “speech perception” passive listening task to the occurrence of each feature. Nine features were consonants defined by a unique combination of place (labial, coronal, and dorsal) and manner (plosive, fricative, nasal) of articulation. Four features were defined by vowel type (low front, low back, high front, high back). The last three features included sentence onsets, obstruent onsets, and onsets defined by the derivative of the acoustic envelope (a surrogate for acoustic onsets, which we previously showed strongly drive neural activity; Hamilton et al., 2018). All trials of a particular speech feature were aligned to the onset of the feature and the median signal was taken across each electrode from −50 to 400 ms. The number of trials of each feature varied depending on how frequently the feature occurred in the TIMIT corpus and on how many recording blocks the subject performed. The mean number of trials per feature in a given subject was 3,223 (range 385–13,777). An exemplar median HGP spatiotemporal response to sentence onsets in the STG is shown in Figure 1A.

Finding canonical patterns of activity during rest is made difficult by the fact that there are no specific external markers to which neural activity may be locked. Thus, to find repeatable patterns of activity during rest, we performed a spatiotemporal principal component analysis (stPCA) on the HGP signals collected during the “resting state” condition (see Supplementary Information—Spatiotemporal PCA for details). In order to maximize the interpretability of the results, the number of principal components was constrained by performing stPCA individually on five anatomical regions of interest defined by the anatomic labeling procedure described in section Electrode Localization (STG, MTG, IFG, SMC, and SMG) rather than on all 256 electrodes together. This was done for each participant separately. The stPCA was trained on the entire resting state block of data, with observations consisting of 300 ms epochs of HGP signal from each electrode in the region. This epoch length was chosen based on inspection of the high gamma responses seen in the “passive listening” condition; a 300 ms epoch was of optimal length to capture the dynamics of the high gamma response to speech features (Figure S2). An exemplar RS spatiotemporal principal component from the STG is shown in Figure 1B, along with its projection onto the participant's brain surface reconstruction at a single time point (Figure 1C).

The correlation coefficient between the median HGP spatiotemporal responses to 16 speech features and the top 50 resting state spatiotemporal principal components (stPC) from each brain region was calculated. This was done for temporal lags of 50–120 ms after the speech feature onset; for each comparison, the lag which maximized the correlation was selected. Statistical significance was determined by comparison against a null distribution of correlations created using temporally shuffled stPCs (200 iterations), using an alpha of 0.001 with Bonferroni correction for multiple comparisons (16 features × 50 stPCs). A correlation coefficient magnitude threshold of 0.1 was also applied in order to eliminate significant but weak correlations. Hierarchical clustering was applied to the composite results from all subjects to look for potential patterns in rest activity that related to speech feature tuning (see Supplementary Information—Hierarchical Clustering for details). For each cluster, the amount of variance in the resting state activity that was explained by the PCs in that cluster was calculated by adding the % variance explained by the individual PCs.

#### Spectrally rotated speech control

In order to control for the possibility that correlations between RS patterns and stimulus evoked patterns are not specific to speech, but rather related to processing of acoustic stimuli more generally, we performed a non-speech control experiment in 3 subjects. These subjects passively listened to a subset of 10 sentences from the same TIMIT speech corpus, but which had been spectrally rotated (described in Hamilton et al., 2018, using algorithms defined in Blesser, 1972). The HGP was aligned and the median response across trials was calculated using the same time-points as determined from the linguistic transcription of the normal, un-rotated TIMIT speech corpus. In this way, the median HGP responses calculated for each of the 16 speech features were actually the responses to the spectrally-rotated versions of those same 16 speech features allowing a direct comparison of their correlations with RS activity. The spectrally rotated speech stimulus has a similar temporal envelope to the original speech, therefore the “sentence onset” and “envelope onset” features are preserved. However, phonological features (manner of articulation, place of articulation, etc.) are destroyed to varying degrees. The same analysis as described above, correlating spatiotemporal responses (to speech features after spectral rotation) with resting state stPCs was performed.

#### Button press control

In order to control for the possibility that correlations between “speech perception” and “resting state” HGP structure in STG are independent of task and specific to the functional role of the STG, two subjects performed a button press motor control task in the absence of any acoustic stimuli, designed to primarily engage the SMC. This task was analyzed as described above (section Spatiotemporal Correlation of Spontaneous and Speech Evoked HGP in STG), with signals from the button press task aligned to the button press event in order to compute the median spatiotemporal response. This 2-dimensional matrix was then correlated with the RS as above.

## Results

### Spontaneous and speech evoked neural activity patterns are correlated in STG

Comparison of the spatiotemporal structure of spontaneous high-gamma activity in STG and the activity evoked by 16 different speech features revealed significant correlations between the two in all subjects. The significant correlations between stPCs trained on spontaneous neural data and speech feature evoked neural activity patterns are shown in Figure 1D. In the next section, we describe the relationship between this spontaneous neural activity and tuning for specific acoustic-phonetic features.

### Spontaneous STG activity relates to distinct acoustic features

Hierarchical clustering revealed 5 groups of resting state spatiotemporal patterns across subjects (Figure 1D). The most robust group was present in all 8 subjects, and showed correlations primarily with sentence onset activity patterns (Videos S1–S3). A mean of 7.8% (range 1.8–14.6%) of RS variance was explained by this group. The second most robust group consisted of patterns correlating with the response to both onsets and consonants, but not vowels (Video S4). This was observed in all left hemisphere subjects (L5–L8) and two right hemisphere subjects (R1 and R2). A mean of 2.5% (0–8%) of the RS variance was accounted for in each patient by this group of activity patterns. A third group consisted of patterns related to onsets and vowels, and explained a mean of 4.9% (0–21.4%) of the RS variance in a given subject (Video S5). Again, this was observed in all left hemisphere subjects (L5–L8) and one right hemisphere subject (R2). A fourth group (present in subjects R1, L5, L7, and L8) showed significant correlation with the responses to all 16 speech features and explained a mean of 4.2% (0–15.5%) of the resting state variance in each subject (Videos S6, S7). Finally, a small group of subjects (R4, L6, and L7) had resting state activity significantly correlated with patterns evoked by plosives. A mean of 3.7% (0–19.1%) of RS variance was explained by this group. In each subject, these spatiotemporal patterns accounted for 16.1–33.5% (mean 21.9%) of the total resting state variance (Figure 1E).

### Structured activity during rest is specific to brain region and function

While the structure of resting state and speech evoked neural activity was correlated in the STG, this was not observed as robustly for the other 4 brain regions investigated (MTG, IFG, SMC, and SMG). These regions showed fewer or no speech evoked high-gamma responses and also had fewer significant correlations between their spontaneous activity and their median HGP activity aligned to speech feature onset (Figure S3). This supports that the correlation observed in STG between RS and speech perception neural activity is related to its functional role in acoustic processing and not due to intrinsic correlations unrelated to functional specialization by brain region. Correlated structure between resting state and motor task aligned activity in STG was not observed in the button press control task (Figure S4), further supporting that the structure of spontaneous activity in STG is specifically related to acoustic processing.

**Figure 2:**
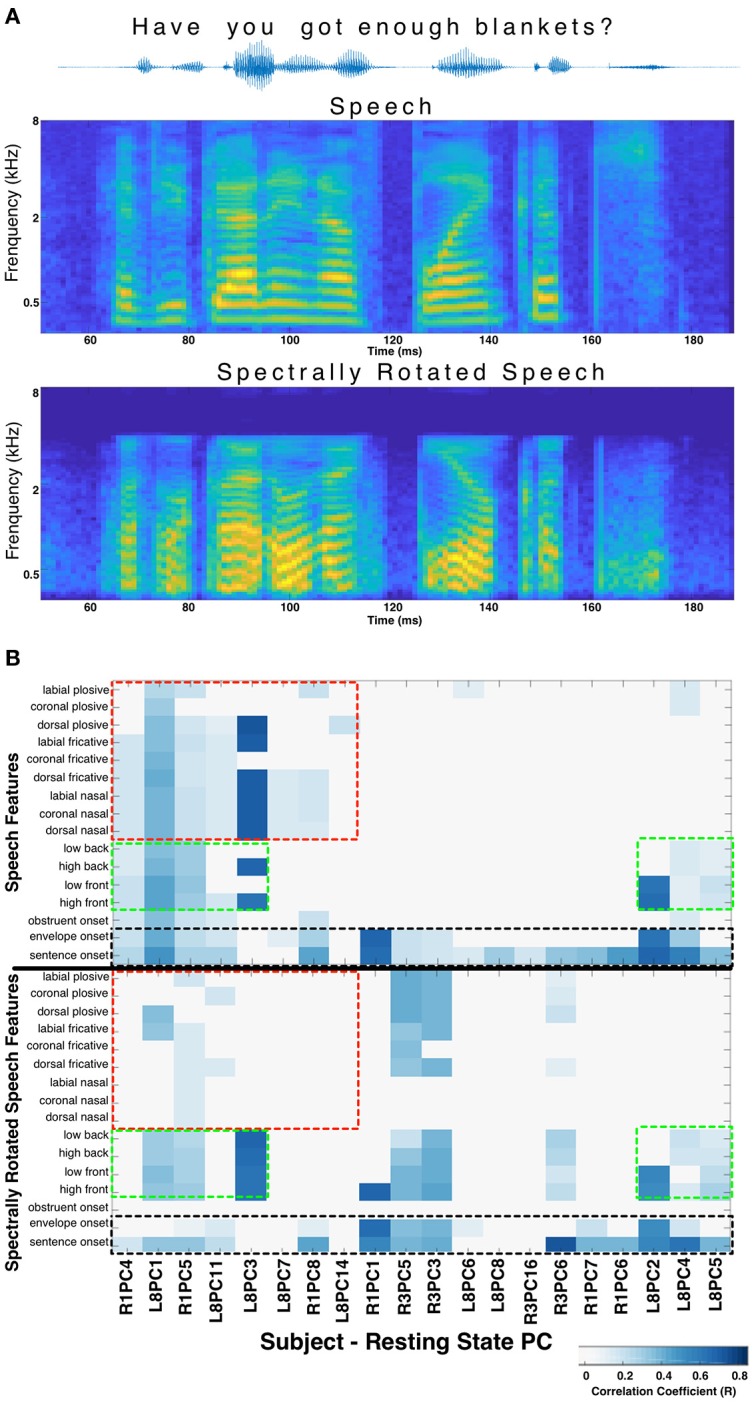
Spectral rotation control. **(A)** The acoustic waveform, spectrogram, and spectrally rotated spectrogram is shown for an example sentence from the TIMIT corpus. In panel **(B)** The significant correlations between resting state stPCs and speech feature evoked HGP responses are compared for normal speech (top) and spectrally rotated speech (bottom) (data from subjects R1, R3, and L8). The y-axis labels in the bottom plot refer to the spectrally rotated version of those speech features, which were defined in the non-rotated TIMIT speech. The result is loss of correlations with consonants (red boxes), while correlations with vowels (green boxes) and onsets (black boxes) are largely unchanged. New correlations introduced by spectral rotation (primarily in Subject R3) are difficult to interpret and were excluded for clarity.

When the phonological features of speech were altered by spectral rotation, changes were observed in the correlations between RS and stimulus evoked activity. Specifically, speech features heavily degraded by the spectral rotation no longer evoked HGP patterns that correlated with the RS patterns. Meanwhile, for features such as onsets, which are less affected by spectral rotation, the correlations with the resting state patterns were largely preserved (Figure 2). This suggests that certain correlations between RS structure and speech perception activity cannot be explained purely by a response pattern to any acoustic stimulus. There appears to be RS structure related to perception of salient acoustic features of speech such as onsets, consonants, and/or vowels.

## Discussion

Spontaneous neural activity has been shown to reflect functional brain organization across many scales and brain regions (Fukushima et al., 2012; Lewis et al., 2016). Resting state structure is thought to support the maintenance of functional brain networks, and may also reflect interactions between individual identity and specific tasks (Gratton et al., 2018). In this study, we hypothesized that the spontaneous activity of the STG, an area known to be involved in acoustic processing and speech perception, would reflect a spatiotemporal organization correlated with the active perception of speech. In all subjects, we found RS activity patterns that were highly correlated with acoustic onset evoked neural activity patterns. These patterns accounted for 7.8% of the RS variance, on average, and occurred in both hemispheres. In a majority of subjects, we also found RS patterns correlating with consonants, vowels, or both. This finding was most robust in the language dominant hemisphere (see Figures 1D,E). On average, these spontaneous activity patterns accounted for 21.9% of the total variance in a subject's resting state activity.

Spatiotemporal structure in spontaneous high gamma activity emerges because ensembles of neurons form networks that default to coordinated activity even in the absence of stimuli. However, in the absence of acoustic stimuli the role/importance of these spontaneous patterns is difficult to determine. One possible explanation is that connectivity patterns during rest strengthen synaptic connections and may be advantageous for rapid detection and processing of future stimuli. Since detection of new acoustic inputs is a fundamental role of the pSTG (Hamilton et al., 2018), it is not surprising that onset responses were most robustly recapitulated in the spontaneous activity. Studies have shown improved speech comprehension and processing of acoustic stimulus trains occurs when there is coherence between the pSTG neural activity and the stimulus envelope (Ahissar et al., 2001; Elhilali et al., 2009). We speculate that the onset response pattern may be adaptive for resetting the ongoing spontaneous activity and rapidly recruiting a large area of pSTG neurons to get in sync with a new stimulus train. Our observation that this was a dominant activity pattern in both language dominant and non-dominant hemispheres further supports its fundamental importance.

Resting state activity also recapitulated speech specific patterns such as responses to consonants and plosives. This spontaneous structure occurred primarily in the language dominant hemisphere (4 of 4 left hemisphere subjects, 2 of 4 left hemispheres subjects) and did not correlate with STG responses to non-speech. We postulate that this structured spontaneous activity is adaptive for recognizing human speech or acquisition of language skills for numerous reasons. First, recognition and processing of human speech must occur without warning and on rapid timescales. By operating in “stand-by” during resting state or before any stimulus is present, the neural networks of speech perception may more efficiently recognize and appropriately process an incoming speech stimulus. Second, infants must develop the neural machinery to perceive and process phonological features as a critical part of learning to process and produce speech. While the structure of spontaneous STG activity in the newborn is unknown, the creation of and maintenance of networks specialized for acoustic feature processing is a likely mechanism for the transition of a newborn from a phonemic universalist to a specialist for a native language. Similarly, in the acquisition of a second language, the structure of spontaneous activity would be expected to evolve as the underlying neural synapses were adapted for perceiving new phonemes with practice. This latter scenario presents a possible experimental model in which to test these hypotheses.

There are several important limitations to the present study. Our sample size of 8 subjects (4 right hemisphere, 4 left hemisphere), limits the strengths of our findings, in particular those findings pertaining to lateralization, which will need to be confirmed in other subjects. A second, intrinsic limitation to our study is the electrode coverage, which is determined by the subject's clinical situation. While onset response patterns were detected in all 8 subjects, we observed differences between subjects with regard to speech specific patterns. The differential neural responses between speech features (e.g., plosives vs. fricatives) are subtle compared to responses to speech versus silence. The former involves modest activation differences on a few electrodes, while the latter involves large activation of a majority of the posterior STG. Thus electrode placement, with 4 mm spacing between electrodes, likely accounted for some of the differences seen between subjects, in addition to inter-subject variability in STG organization.

We did not control for the possibility of “internal speech” or subjects activating their STG during resting state by thinking in their own language. Future studies could attempt to control for this possibility by analyzing data from non-REM sleep. Finally, we included a motor task (button-press) and spectrally rotated speech as controls, however additional experiments with other non-speech stimuli are needed to better understand the resting state structure of STG.

In this study, we present evidence that the RS structure of STG activity robustly recapitulates its task-evoked response to acoustic onsets. Further, secondary patterns in RS activity appear to correlate with task-evoked responses to speech features. These findings are consistent with the task related RS structure previously seen in visual and auditory cortices of macaques (Fukushima et al., 2012; Lewis et al., 2016). The role of these spontaneous spatiotemporal activity patterns remains to be elucidated.

## Author contributions

JB, LH, and EC: experimental design; data collection, manuscript preparation; JB and LH: data analysis.

### Conflict of interest statement

The authors declare that the research was conducted in the absence of any commercial or financial relationships that could be construed as a potential conflict of interest.

## Supplementary material

Video S1 The evoked spatiotemporal response to envelope onsets (left) and a significantly correlated spatiotemporal principal component (right) are shown side-by-side for Subject R1. The correlation strength is *R* = 0.68. The evoked response begins with a 50 ms lag after the stimulus presentation because only the 300 ms epoch which maximized R is shown. In both the envelope onset response and the resting state stPC, an increase in high gamma power can be observed in a discrete region of middle to posterior STG.

Video S2 The evoked spatiotemporal response to envelope onsets (left) and a significantly correlated spatiotemporal principal component (right) are shown side-by-side for Subject L5. The correlation strength is *R* = 0.58. In both the envelope onset response and the resting state stPC, an increase in high gamma power can be observed in a discrete region of middle to posterior STG. Areas of negative HGP modulations in the anterior STG and far posterior STG correspond with areas of lower stPC coefficient weights in the resting state.

Video S3 The evoked spatiotemporal response to sentence onsets (left) and a significantly correlated spatiotemporal principal component (right) are shown side-by-side for Subject R2. The correlation strength is *R* = 0.45. In both the sentence onset response and the resting state stPC, an early negative modulation of high gamma power in anterior STG is observed, followed by a later increase in high gamma power in posterior STG sites.

Video S4 The evoked spatiotemporal response to dorsal plosives (left) and a significantly correlated spatiotemporal principal component (right) are shown side-by-side for Subject L7. The correlation strength is *R* = 0.78. In both the dorsal plosive response and the resting state stPC, a wide area of the posterior STG is activated, with a spatiotemporal pattern that appears as a strong increase in high gamma power posteriorly, followed by a later response in slightly more anterior areas surrounding this early response.

Video S5 The evoked spatiotemporal response to sentence onsets (left) and a significantly correlated spatiotemporal principal component (right) are shown side-by-side for Subject L8. The correlation strength is *R* = 0.36. In both the sentence onset response and the resting state stPC, an increase in high gamma power can be observed in a discrete region of middle to posterior STG. In the resting state, there is also negative modulation in far posterior sites, which is not seen in the sentence onset response.

Video S6 The evoked spatiotemporal response to low back vowels (left) is shown side-by-side with a resting state spatiotemporal principal component (right) for Subject L5. The correlation strength is *R* = 0.44. In the stimulus-evoked response as well as the resting state stPC, mid- to posterior STG sites showed an increase in high gamma power, followed by a negative modulation.

Video S7 The evoked spatiotemporal response to high front vowels (left) is shown side-by-side with a resting state spatiotemporal principal component (right) for Subject R1. The correlation strength is *R* = 0.3. In the stimulus-evoked response as well as the resting state stPC, mid- to posterior STG sites showed an increase in high gamma power, though this response spread more posteriorly in the resting state activity. In resting state, a strong negative modulation followed this positive response, while in the evoked-state only a weak negative response was observed following the first positive evoked response.

## References

[B1] Ahissar E. Nagarajan S. Ahissar M. Protopapas A. Mahncke H. Merzenich M. M. (2001). Speech comprehension is correlated with temporal response patterns recorded from auditory cortex. Proc. Natl. Acad. Sci. U.S.A. 98, 13367–13372. 10.1073/pnas.201400998 11698688 PMC60877

[B2] Berezutskaya J. Freudenburg Z. V. Güçlü U. van Gerven M. A. Ramsey N. F. (2017). Neural tuning to low-level features of speech throughout the perisylvian cortex. J. Neurosci. 37, 7906–7920. 10.1523/JNEUROSCI.0238-17.2017 28716965 PMC6596904

[B3] Blesser B. (1972). Speech perception under conditions of spectral transformation: I. Phonetic characteristics. J. Speech Lang. Hear. Res. 15, 5–41. 10.1044/jshr.1501.05 5012812

[B4] Breshears J. D. Roland J. L. Sharma M. Gaona C. M. Freudenburg Z. V. Tempelhoff R.. (2010). Stable and dynamic cortical electrophysiology of induction and emergence with propofol anesthesia. Proc. Natl. Acad. Sci. U.S.A. 107, 21170–21175. 10.1073/pnas.1011949107 21078987 PMC3000270

[B5] Chang E. F. Rieger J. W. Johnson K. Berger M. S. Barbaro N. M. Knight R. T. (2010). Categorical speech representation in human superior temporal gyrus. Nat. Neurosci. 13, 1428–1432. 10.1038/nn.2641 20890293 PMC2967728

[B6] Chelaru M. I. Hansen B. J. Tandon N. Conner C. R. Szukalski S. Slater J. D.. (2016). Reactivation of visual-evoked activity in human cortical networks. J. Neurophysiol. 115, 3090–3100. 10.1152/jn.00724.2015 26984423 PMC4946592

[B7] Elhilali M. Ma L. Micheyl C. Oxenham A. J. Shamma S. A. (2009). Temporal coherence in the perceptual organization and cortical representation of auditory scenes. Neuron 61, 317–329. 10.1016/j.neuron.2008.12.005 19186172 PMC2673083

[B8] Foster B. L. Rangarajan V. Shirer W. R. Parvizi J. (2015). Intrinsic and task-dependent coupling of neuronal population activity in human parietal cortex. Neuron 86, 578–590. 10.1016/j.neuron.2015.03.018 25863718 PMC4409557

[B9] Friederici A. D. (2012). The cortical language circuit: from auditory perception to sentence comprehension. Trends Cogn. Sci. 16, 262–268. 10.1016/j.tics.2012.04.001 22516238

[B10] Fukushima M. Saunders R. C. Leopold D. A. Mishkin M. Averbeck B. B. (2012). Spontaneous high-gamma band activity reflects functional organization of auditory cortex in the awake macaque. Neuron 74, 899–910. 10.1016/j.neuron.2012.04.014 22681693 PMC3372858

[B11] Garofolo J. S. Lamel L. F. Fisher W. M. Fiscus J. G. Pallett D. S. (1993). DARPA TIMIT Acoustic-Phonetic Continous Speech Corpus CD-ROM. NIST Speech Disc 1-1.1. NASA STI/Recon Technical Report n 93.

[B12] Gratton C. Laumann T. O. Nielsen A. N. Greene D. J. Gordon E. M. Gilmore A. W. (2018). Functional brain networks are dominated by stable group and individual factors, not cognitive or daily variation. Neuron 98, 439–452.e435. 10.1016/j.neuron.2018.03.035 29673485 PMC5912345

[B13] Hamilton L. Edwards E. Chang E. (2018). A spatial map of onset and sustained responses to speech in the human superior temporal gyrus. Curr. Biol. 28, 1860–1871.e4. 10.1016/j.cub.2018.04.033 29861132

[B14] Hamilton L. S. Chang D. L. Lee M. B. Chang E. F. (2017). Semi-automated anatomical labeling and inter-subject warping of high-density intracranial recording electrodes in electrocorticography. Front. Neuroinformatics 11:62. 10.3389/fninf.2017.00062 29163118 PMC5671481

[B15] He B. J. Snyder A. Z. Zempel J. M. Smyth M. D. Raichle M. E. (2008). Electrophysiological correlates of the brain's intrinsic large-scale functional architecture. Proc. Natl. Acad. Sci. U.S.A. 105, 16039–16044. 10.1073/pnas.0807010105 18843113 PMC2564983

[B16] Leaver A. M. Rauschecker J. P. (2010). Cortical representation of natural complex sounds: effects of acoustic features and auditory object category. J. Neurosci. 30, 7604–7612. 10.1523/JNEUROSCI.0296-10.2010 20519535 PMC2930617

[B17] Lewis C. M. Bosman C. A. Womelsdorf T. Fries P. (2016). Stimulus-induced visual cortical networks are recapitulated by spontaneous local and interareal synchronization. Proc. Natl. Acad. Sci. U.S.A. 113, E606–E615. 10.1073/pnas.1513773113 26787906 PMC4747694

[B18] Mesgarani N. Cheung C. Johnson K. Chang E. F. (2014). Phonetic feature encoding in human superior temporal gyrus. Science 343, 1006–1010. 10.1126/science.1245994 24482117 PMC4350233

[B19] Moses D. A. Mesgarani N. Leonard M. K. Chang E. F. (2016). Neural speech recognition: continuous phoneme decoding using spatiotemporal representations of human cortical activity. J. Neural Eng. 13:056004. 10.1088/1741-2560/13/5/056004 27484713 PMC5031534

[B20] Steinschneider M. Nourski K. V. Kawasaki H. Oya H. Brugge J. F. Howard M. A. III. (2011). Intracranial study of speech-elicited activity on the human posterolateral superior temporal gyrus. Cereb. Cortex 21, 2332–2347. 10.1093/cercor/bhr014 21368087 PMC3169661

[B21] Tang C. Hamilton L. Chang E. (2017). Intonational speech prosody encoding in the human auditory cortex. Science 357, 797–801. 10.1126/science.aam8577 28839071 PMC9584035

[B22] Vincent J. L. Patel G. H. Fox M. D. Snyder A. Z. Baker J. T. Van Essen D. C.. (2007). Intrinsic functional architecture in the anaesthetized monkey brain. Nature 447, 83–86. 10.1038/nature05758 17476267

