# Supplementary material for: Spontaneous Neural Activity in the Superior Temporal Gyrus Recapitulates Tuning for Speech Features
Source: Front Hum Neurosci. 2018 Sep 18;12:360. doi: 10.3389/fnhum.2018.00360 (PMC6153351; doi:10.3389/fnhum.2018.00360)

Supplementary Material

Spontaneous neural activity in the superior temporal gyrus recapitulates tuning for speech features

Jonathan D. Breshears, M.D., Liberty S. Hamilton, Ph.D., Edward F. Chang, M.D.*

Correspondence:
Edward F. Chang, MD.
edward.chang@ucsf.edu

Supplementary Figures and Tables

SI Figure 1. The electrode coverage for each subject is plotted on their pial surface reconstruction. Electrodes excluded from the analysis due to artifact are not shown. Classification by brain region is indicated by color.

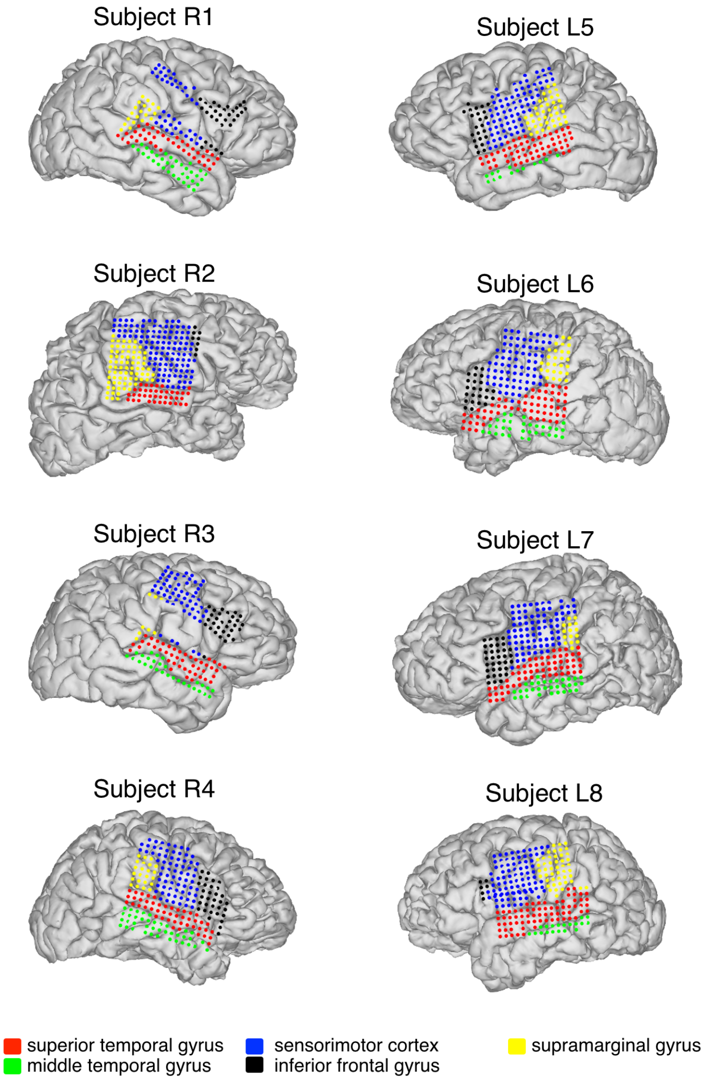

SI Figure 2. The median HGP response to all 16 speech features from STG electrodes is shown for two exemplar subjects (R1 & L7). A 300ms epoch was chosen for the spatiotemporal PCA because it reasonably captured the rising and falling dynamics of the HGP response after speech feature onset (time = 0).

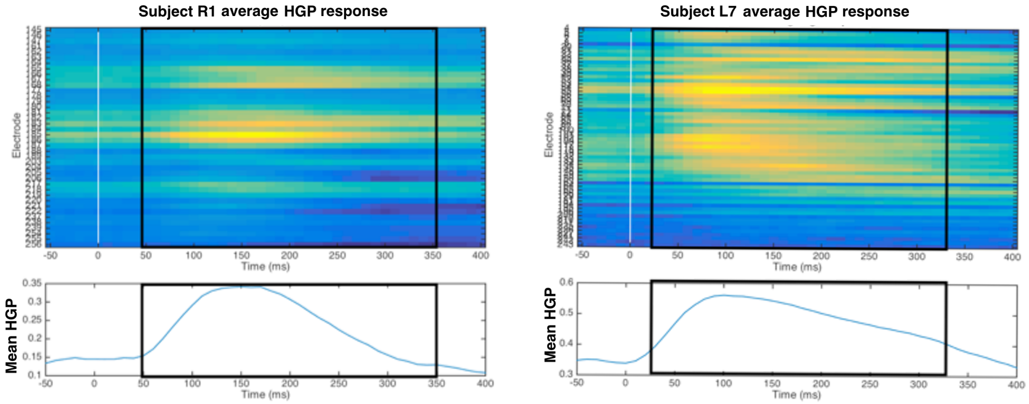

Spatiotemporal PCA (stPCA)

After being z-scored individually as described in section **2.2.1 Neural Recordings**, all individual resting state (RS) blocks from a given patient were concatenated together to create the 2D matrix X with dimensions of [time-points x electrodes] or "time" and "space" (**Eqn 1**).

Equation 1

electrodes →

$$X = \begin{bmatrix} n_{1,1} & n_{2,1} & n_{3,1} & n_{4,1} \cdots & n_{e,1} \\ n_{1,2} & n_{2,2} & n_{3,2} & \cdots & \vdots \\ n_{1,3} & n_{2,3} & n_{2,3} & \cdots & \vdots \\ n_{1,4} & \vdots & \vdots & \ddots & \vdots \\ \vdots & \vdots & \vdots & & \vdots \\ n_{1,t} & \cdots & \cdots & \cdots & n_{e,t} \end{bmatrix}$$

timepoints ↓

Where *n* is a normalized high gamma power value on electrode *e* at timepoint *t*. The analysis was performed on 100 Hz data, therefore each sample *n* represents 10ms.

Spatiotemporal PCA was trained on the entire set of resting state data by restructuring matrix X into a time-lagged matrix, $X_{spatiotemporal}$, with dimensions of [time-points x (electrodes & time-lags)]. This was done by iteratively shifting X backwards by 10ms (one time-lag) and concatenating it with itself 30 times (10ms x 30 = 300 ms), as shown in **Eqn 2.**

Equation 2

$$X_{spatiotemporal} = \begin{bmatrix} n_{1,1} & n_{2,1} & n_{3,1} \cdots n_{e,1} & n_{1,2} & n_{2,2} & n_{3,2} \cdots n_{e,2} & n_{1,3} & n_{2,3} & n_{3,3} \cdots n_{e,3} & & n_{1,30} & n_{2,30} & n_{3,30} \cdots n_{e,30} \\ n_{1,2} & n_{2,2} & n_{3,2} \cdots \vdots & n_{1,3} & n_{2,3} & n_{2,3} \cdots \vdots & n_{1,4} & n_{2,4} & n_{3,4} \cdots n_{e,4} & & n_{1,31} & n_{2,31} & n_{3,31} \cdots n_{e,31} \\ n_{1,3} & n_{2,3} & n_{2,3} \cdots \vdots & n_{1,4} & n_{2,4} & n_{3,4} \cdots \vdots & \vdots & \vdots & \ddots \; \vdots & \cdots & \vdots & \vdots & \ddots \\ \vdots & \vdots & \ddots \; \vdots & \vdots & \vdots & \ddots \; \vdots & \vdots & \vdots & \ddots \; \vdots & & \vdots & \vdots & \ddots \\ n_{1,(t-30)} & n_{2,(t-30)} & n_{3,(t-30)} \cdots n_{e,(t-30)} & n_{1,(t-29)} & n_{2,(t-29)} & n_{3,(t-29)} \cdots n_{e,(t-29)} & n_{1,(t-28)} & n_{2,(t-28)} & n_{3,(t-28)} \cdots n_{e,(t-28)} & & n_{1,t} & n_{2,t} & n_{3,t} \cdots n_{e,t} \end{bmatrix}$$

Each row of $X_{spatiotemporal}$ is an observation of the HGP at each of *e* electrodes over a 300ms epoch (30 time-lags). Adjacent rows are spatiotemporal observations shifted forward or backward in time by 10ms. PCA was performed on $X_{spatiotemporal}$ in using the built-in MATLAB function *pca()* in order to obtain the principal component coefficients. Once obtained, the matrix of coefficients for each principal component, denoted *PC*, was restructured from a 1-dimentional vector of electrodes and time-lags, back into a 2D matrix $PC_{spatiotemporal}$ with dimensions of [time-points x electrodes] as illustrated in **Eqn 3.**

Equation 3

$$PC = \begin{bmatrix} w_{1,1} \\ w_{2,1} \\ w_{3,1} \\ \vdots \\ w_{e,1} \\ w_{1,2} \\ w_{2,2} \\ \vdots \\ w_{e,2} \\ w_{1,3} \\ w_{2,3} \\ \vdots \\ w_{e,3} \\ w_{1,4} \\ w_{2,4} \\ \vdots \\ w_{e,30} \end{bmatrix} \Rightarrow PC_{spatiotemporal} = \begin{bmatrix} w_{1,1} & w_{2,1} & w_{3,1} \cdots & w_{e,1} \\ w_{1,2} & w_{2,2} & w_{3,2} \cdots & w_{e,2} \\ w_{1,3} & w_{2,3} & w_{3,3} \cdots & w_{e,3} \\ \vdots & \vdots & \vdots \quad \ddots & \vdots \\ w_{1,30} & w_{2,30} & w_{3,30} \cdots & w_{e,30} \end{bmatrix}$$

Hierarchical Clustering

Hierarchical clustering was performed using built-in MATLAB functions (*linkage*() and *cluster*()). First the [1x16] vectors of significant correlations between the PCs (from every subject) and the 16 speech features were concatenated together and converted to a composite binary matrix (1 for any significant non-zero correlation, 0 otherwise). Next the Euclidean distance was calculated between each of the rows of this matrix (each vector representing the profile of speech feature correlations with a single principal component from a single subject). A linkage tree was then constructed using the inner square distance (or 'ward') method, and finally clusters were constructed using the distance criterion and setting *maxclust* = 5. Five was chosen as the maximum allowed number of clusters based on experimentation with a range of *maxclust* values [2- 8].

SI Figure 3. The median high gamma power response to labial plosives from all 5 brain regions investigated is shown for eight subjects in the panels of (**A**). A robust response is uniformly seen in the STG. Some neighboring regions show responses to the acoustic stimulus on a few electrodes that border the STG; due to the margin of error in electrode localization, it is possible that these electrodes are actually recording from the STG. A similar pattern was seen for all 16 speech features. (**B**) shows the significant correlations between the resting state stPCs and the speech feature responses in 5 brain regions for all eight subjects. STG had the most robust correlations, followed by SMC, MTG, SMG, and IFG.

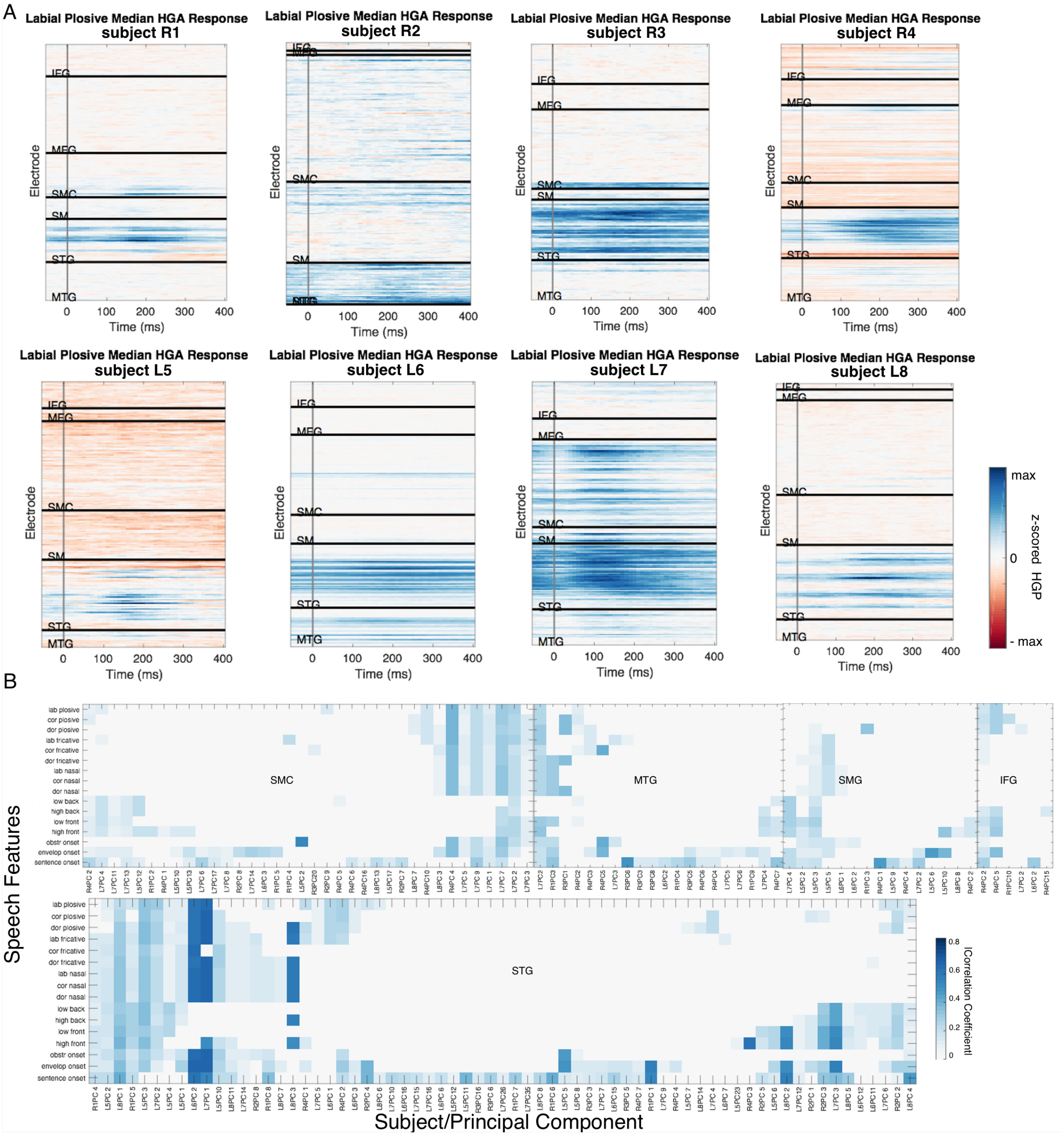

SI Figure 4. The median high gamma power response to a button press is shown for subjects R1 and L8. Button press occurred at time = 0. As expected, there was no large response from STG, while some response is seen in SMC and IFG. The minimal response seen in SMC is explained by the electrode coverage, which did not extend superiorly enough to record a robust hand motor response. There were no significant correlations found between resting state stPCs and the spatiotemporal responses to the button press in any brain region. This was true for 50-300ms time epochs with lags ranging from 200ms pre-button press to 200ms post-button press.

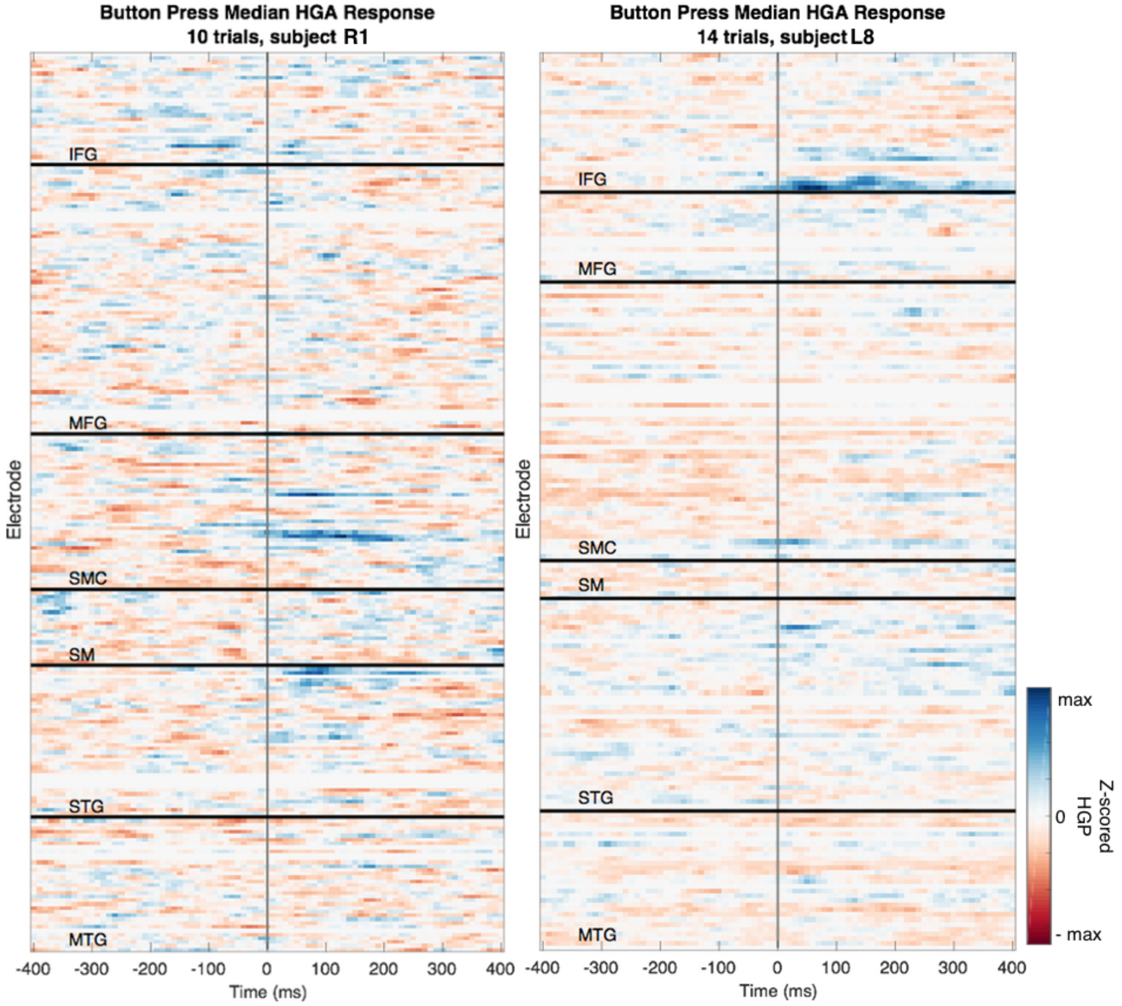

Supplement: Supplementary file 8 [file Data_Sheet_1.pdf]
